# Approaches to Anti-Ageism Interventions

**DOI:** 10.1093/geroni/igaa057.2333

**Published:** 2020-12-16

**Authors:** Tracey Gendron, Jennifer Inker

**Affiliations:** Virginia Commonwealth University, Richmond, Virginia, United States

## Abstract

Ageism, a multidimensional construct, is also understood as a relational process whereby perceptions and behaviors toward older individuals by younger individuals not only damage the self-esteem of elders, but also create a hostile environment for their own future social interactions and their own future self-development as elders. Anti-ageism interventions have the hefty task of improving attitudes and behaviors toward aging within all of these contexts. This presentation will discuss findings from two different anti-ageism interventions both designed to mitigate the negative impacts of ageism. Results from a study on an intergenerational arts-based program found that after participation students demonstrated a positive change in their attitudes toward older adults. Findings from a video-based ageism intervention among a sample of 265 staff members in 15 senior living communities demonstrated decreased internalized aging anxiety as well as decreased ageist behaviors directly after the training and at three month post follow-up. Given the complex and systemic nature of ageism, diversity is necessary in scope and type of intervention in order to reach the broadest audience.

